# Primary care physicians’ perceptions of barriers and facilitators to management of chronic kidney disease: A mixed methods study

**DOI:** 10.1371/journal.pone.0221325

**Published:** 2019-08-22

**Authors:** C. John Sperati, Sandeep Soman, Varun Agrawal, Yang Liu, Khaled Abdel-Kader, Clarissa J. Diamantidis, Michelle M. Estrella, Kerri Cavanaugh, Laura Plantinga, Jane Schell, James Simon, Joseph A. Vassalotti, Michael J. Choi, Bernard G. Jaar, Raquel C. Greer, on behalf of the National Kidney Foundation Education Committee

**Affiliations:** 1 Division of Nephrology, Department of Medicine, Johns Hopkins University School of Medicine, Baltimore, Maryland, United States of America; 2 Division of Nephrology, Henry Ford Hospital, Detroit, Michigan, United States of America; 3 Division of Nephrology and Hypertension, University of Vermont College of Medicine, Burlington, Vermont, United States of America; 4 Johns Hopkins Medicine International, Johns Hopkins Medical Institutions, Baltimore, Maryland, United States of America; 5 Division of Nephrology and Hypertension, Department of Medicine, Vanderbilt University Medical Center; Vanderbilt Center for Kidney Disease, Nashville, Tennessee, United States of America; 6 Divisions of General Internal Medicine and Nephrology, Duke University School of Medicine, Durham, North Carolina, United States of America; 7 Kidney Health Research Collaborative, Department of Medicine, University of California, San Francisco and San Francisco VA Health Care System, San Francisco, California, United States of America; 8 Department of Medicine, Emory University, Atlanta, Georgia, United States of America; 9 Section of Palliative Care and Medical Ethics, Division of Renal-Electrolyte, University of Pittsburgh Medical Center, Pittsburgh, Pennsylvania, United States of America; 10 Department of Nephrology and Hypertension, Glickman Urologic and Kidney Institute, Cleveland Clinic, Cleveland, Ohio, United States of America; 11 Icahn School of Medicine at Mount Sinai, New York, NY, United States of America; 12 National Kidney Foundation, New York, New York, United States of America; 13 Nephrology Center of Maryland, Baltimore, Maryland, United States of America; 14 The Welch Center for Prevention, Epidemiology, and Clinical Research, Baltimore, Maryland, United States of America; 15 Division of General Internal Medicine, Department of Medicine, Johns Hopkins University School of Medicine, Baltimore, Maryland, United States of America; Imperial College London, UNITED KINGDOM

## Abstract

**Background:**

Given the high prevalence of chronic kidney disease (CKD), primary care physicians (PCPs) frequently manage early stage CKD. Nonetheless, there are challenges in providing optimal CKD care in the primary care setting. This study sought to understand PCPs’ perceptions of barriers and facilitators to the optimal management of CKD.

**Study design:**

Mixed methods study

**Settings and participants:**

Community-based PCPs in four US cities: Baltimore, MD; St. Louis, MO; Raleigh, NC and San Francisco, CA.

**Methodology:**

We used a self-administered questionnaire and conducted 4 focus groups of PCPs (n = 8 PCPs/focus group) in each city to identify key barriers and facilitators to management of patients with CKD in primary care.

**Analytic approach:**

We conducted descriptive analyses of the survey data. Major themes were identified from audio-recorded interviews that were transcribed and coded by the research team.

**Results:**

Of 32 participating PCPs, 31 (97%) had been in practice for >10 years, and 29 (91%) practiced in a non-academic setting. PCPs identified multiple barriers to managing CKD in primary care including at the level of the patient (e.g., low awareness of CKD, poor adherence to treatment recommendations), the provider (e.g., staying current with CKD guidelines), and the health care system (e.g., inflexible electronic medical record, limited time and resources). PCPs desired electronic prompts and lab decision support, concise guidelines, and healthcare financing reform to improve CKD care.

**Conclusions:**

PCPs face substantial but modifiable barriers in providing care to patients with CKD. Interventions that address these barriers and promote facilitative tools may improve PCPs’ effectiveness and capacity to care for patients with CKD.

## Introduction

Patients with chronic kidney disease (CKD) are medically complex, with a high likelihood of significant comorbidity and an increased risk of progression to end stage renal disease and death.[1, 2] Recognized as a public health priority, CKD ranks 18^th^ in causes of death worldwide in 2010 and 9^th^ in the United States in 2015.[3, 4] The past 20 years has witnessed an 82% increase in deaths worldwide due to kidney disease, an increase similar to that of diabetes mellitus and greater than cancer.[3] Moreover, the prevalence of CKD in the United States is estimated at 15% of the non-institutionalized adult population, corresponding to over 2000 patients per U.S. nephrology provider.[5, 6] In light of the sizeable at-risk population, primary care physicians (PCPs) may have to provide the bulk of care for patients with early (Stage 1–3) CKD. PCPs, however, have been shown to suboptimally recognize and manage patients with CKD.[7–11] This mixed methods study sought to better understand PCPs’ perceptions of patient-, provider-, and systems-level barriers to CKD management in the primary care setting. In addition, we sought PCPs’ views on potential facilitators to high quality CKD care delivery.

## Materials and methods

Using a mixed-methods approach, we conducted a self-administered survey and four qualitative focus groups of 32 PCPs in four U.S. cities (Baltimore, MD, St. Louis, MO, Raleigh, NC and San Francisco, CA) (n = 8 PCPs/focus group in each city) to identify 1) PCPs’ perceived barriers to the care of patients with CKD, and 2) PCPs’ views of potential tools and resources which could improve their care of patients with CKD. Both qualitative and quantitative data were used to obtain a deeper, broader understanding of the challenges PCPs experience in the provision of CKD care and to increase the validity of the findings.

PCPs from each city were purposively recruited by Baltimore Research (Towson, MD) to participate. Baltimore Research recruits providers from an internal list of physicians who have previously opted to participate in research studies. To be eligible for participation, respondents needed to have at least one half day clinic per week, see >40 patients per month, spend the majority of their clinical time delivering outpatient primary care, and provide care for patients with CKD. Since PCPs from different specialties (i.e., family practice and internal medicine), practice settings (i.e., community-based and university/medical school-based practice), genders (i.e., male and female), and racial/ethnic populations (i.e., included PCPs identifying as Black/African American, Hispanic/Latino, Native American, Native Hawaiian or Other Pacific Islander) may perceive different barriers to delivery of optimal CKD care, we pre-determined that each focus group would have at least 1–2 PCPs from each of the above specified groups. We selected a sample size (32 participants, 4 focus groups) expected to provide adequate saturation of major themes. All participants were informed of the overall goal of the study and written consent was obtained. The study protocol was approved by The Johns Hopkins Medicine Institutional Review Board. We did not systematically gather information from providers who received an invitation from Baltimore Research but did not participate in the study.

Each 90-minute focus group session was conducted between April and June 2015 at Baltimore Research or affiliated location. Data on physicians’ demographic and practice characteristics, comfort with managing and educating patients with CKD, and access to clinical management tools were obtained via a brief self-administered questionnaire (S1 Appendix) developed by a team with clinical expertise in CKD. Questions featured a 5 point Likert scale ranging from strongly agree to strongly disagree, and responses were categorized as strongly agree and agree versus neutral, disagree or strongly disagree. The focus group discussion began immediately following completion of the questionnaire. One investigator (RCG), a female general internist and health service researcher with expertise in qualitative methods[9, 12, 13] and no prior relationship with study participants, conducted all sessions using a standardized question guide developed by the National Kidney Foundation Education Committee (RCG, KC, CJD, MME, KA, VA, JAV, MJC, and BGJ). The question guide was further refined by physicians with expertise in primary care, nephrology, and qualitative research methods. The question guide consisted of open-ended questions with the goal of identifying PCPs’ perceived barriers and facilitators to the care of patients with CKD (Box 1). The question guide was refined in an iterative fashion to address emerging themes from prior focus group sessions. The focus group moderator (RCG) took field notes during and after the focus session. The focus groups were audiotaped and transcribed verbatim for thematic content analysis.[14, 15]

**Box 1. Interview guide**

Primary care physician interview questions and probes

Demographics

- Briefly describe your practice setting.
- How long have you been in practice?

Patient characteristics

- Briefly describe your typical patient with CKD.

Approach to CKD screening

- What are the challenges you face in screening patients for CKD?
- Describe resources, tools, or features of your practice that make it easier to screen for CKD.
- What would be helpful in screening patients for CKD?

Experience with managing CKD

- What are the biggest challenges you face in managing patients with CKD?
- Of the following, what are the 2 most challenging and 2 least challenging aspects of care to manage in patients with CKD? Why?
  ○ Diagnosis of CKD
  ○ Evaluation of CKD cause
  ○ Managing risk factors for CKD progression
  ○ Assessment and management of CKD complications
  ○ Medication safety
  ○ Educating patients with CKD
- Describe resources, tools, or practice features that help you address these aspects of care.
- What would be helpful in addressing these aspects of care in patients with CKD?

Tools to facilitate care

- What would you incorporate into your practice to help you care for patients with CKD?

We used descriptive statistics to describe questionnaire responses. We used content analysis to analyze the focus group discussions related to the diagnosis of CKD, evaluation of CKD, management of CKD, risk factors for CKD progression and CKD-related complications, and educating patients about CKD. Two investigators (RCG and YL) independently reviewed the first 2 focus groups transcripts to develop an initial coding scheme using an inductive approach representing the relevant concepts (i.e., codes emerged from the data and were not predefined). [14, 15] Descriptive codes were assigned to segments of text of varying size that describe the challenges or facilitators PCPs experience in caring for their patients with CKD. The investigators then reviewed the initial coding schemes together and agreed upon a final coding scheme that was applied to all transcripts. Any new codes identified from the review of the additional focus group transcripts were reviewed and added to the final coding scheme. The codes were subsequently categorized into a final list of major themes and subthemes regarding PCPs’ perceptions of barriers and facilitators to the care of patients with CKD. The themes were then organized to describe the PCPs’ perceived barriers at the patient, provider, and system-level. Evidence of thematic saturation was noted when no new themes were identified in the fourth focus group discussion. We compared the findings from the quantitative and qualitative data. We informally reviewed our study results with PCPs at our institution and study team members with expertise in the clinical care of CKD. ATLAS.ti version 5.0. (ATLAS.ti GmbH, Berlin, Germany) was used for data management.

## Results

More than half (n = 22, 69%) of the PCP respondents practiced in a single-specialty private practice, with only 9% (n = 3) in a teaching hospital (Table 1). The PCPs had a mean age of 53±8 years and were predominately male (n = 19, 59%) and white (n = 21, 66%). Most possessed an MD degree (versus DO) (n = 30, 94%) and had been in practice for more than 15 years (n = 23; 72%). Respondents spent most of their time in clinical practice (median percent clinical time 98 [IQR 88–100]) and 38% (n = 12) saw more than 100 patients per week. Twenty-two (69%) respondents saw more than 10 patients with CKD per week.

**Table 1:** Characteristics of primary care physicians (N = 32).

Participant characteristics	N (%)
Age, mean years (SD)	53 (8)
Gender	
Male	19 (59)
Female	13 (41)
Race/ethnicity	
White	21 (66)
Black or African American	2 (6)
Hispanic or Latino	2 (6)
Asian	5 (16)
Other	2 (6)
Medical specialty	
Internal medicine	19 (59)
Family practice	13 (41)
Training	
Doctor of Medicine (MD)	30 (94)
Doctor of Osteopathic Medicine (DO)	2 (6)
Practice setting	
Solo private practice	10 (31)
Single specialty group private practice	12 (38)
Multispecialty group practice	5 (16)
University hospital or medical school	2 (6)
Community, teaching hospital	1 (3)
Community, non-teaching hospital	1 (3)
Government health care facility	1 (3)
Percent clinical time	
≥ 80	30 (94)
< 80	2 (6)
Percent clinical time, median (IQR)	98 (88–100)
Percent research time, median (IQR)	0 (0–0)
Percent administrative time, median (IQR)	0 (0–10)
Number of patients per week	
≤ 100	20 (62)
> 100	12 (38)
Number of CKD patients per week	
10 or less	10
11–20	10
21–30	5
31–40	3
> 40	4
Number of years in practice	
0–5	
6–10	1 (3)
11–15	8 (25)
> 15 years	23 (72)
EHR use	
yes, part EMR and part paper	8 (25)
yes, all EMR/yes, part EMR and part paper	1 (3)
yes, all EMR	23 (72)
Report following CKD guidelines^*^	
No	14 (45)
Yes	17 (54)

^*^n = 31

Forty-five percent (n = 14) of PCPs reported that they did not follow CKD guidelines. Table 2 summarizes PCP-reported comfort with managing CKD and the availability of tools to facilitate that care. Although most (n = 27, 84%) strongly agreed or agreed that they felt comfortable managing patients with CKD, many were not comfortable managing specific complications of CKD such as anemia (n = 14, 44%), bone disorders (n = 16, 50%), and metabolic acidosis (n = 22, 69%). PCPs frequently cited a lack of available tools to facilitate management of specific complications of CKD, such as anemia (n = 21, 66%), hyperkalemia (n = 16, 50%), metabolic acidosis (n = 22, 69%), and bone disorders (n = 23, 72%). Similarly, a lack of tools and resources for educating patients was noted for diagnosing CKD, hypertension management, medication risks, anemia, hyperkalemia, bone disorders, and metabolic acidosis (44–78%).

**Table 2:** PCP-reported comfort with managing CKD and access to clinical tools (n = 32).

	N (%)				
Themes	Strongly Agree	Agree	Neutral	Disagree	Strongly Disagree
I feel comfortable:					
making the diagnosis of CKD in my patients	15 (47)	15 (47)	2 (6)	0 (0)	0 (0)
educating my patients about CKD	9 (29)	18 (58)	4 (13)	0 (0)	0 (0)
managing my patients with CKD^*^	8 (26)	19 (61)	4 (13)	0 (0)	0 (0)
managing medication dosing in my patients with CKD	5 (16)	21 (66)	6 (19)	0 (0)	0 (0)
avoiding nephrotoxic medications in my patients with CKD^*^	13 (42)	17 (55)	1 (3)	0 (0)	0 (0)
managing hypertension in my patients with CKD	11 (34)	19 (59)	2 (6)	0 (0)	0 (0)
managing anemia of CKD in my patients	4 (13)	14 (44)	10 (31)	4 (13)	0 (0)
managing bone disorders of CKD in my patients	1 (3)	15 (47)	11 (34)	5 (16)	0 (0)
managing electrolyte disorders in my patients with CKD	3 (9)	18 (56)	8 (25)	2 (6)	1 (3)
managing metabolic acidosis in my patients with CKD	1 (3)	9 (28)	16 (50)	4 (13)	2 (6)
I have available tools which help me to:					
diagnose CKD	8 (25)	16 (50)	5 (16)	3 (9)	0 (0)
manage CKD^*^	6 (19)	16 (52)	6 (19)	3 (10)	0 (0)
manage medication dosing^*^	6 (19)	19 (61)	3 (10)	3 (10)	0 (0)
avoid prescribing nephrotoxic medications	7 (22)	18 (56)	5 (16)	2 (6)	0 (0)
manage hypertension in my patients with CKD	4 (13)	18 (56)	7 (22)	3 (9)	0 (0)
manage anemia of CKD	2 (6)	9 (28)	14 (44)	6 (19)	1 (3)
manage bone disorders of CKD	2 (6)	7 (22)	17 (53)	5 (16)	1 (3)
manage hyperkalemia in CKD^**^	2 (7)	12 (40)	10 (33)	5 (17)	1 (3)
manage metabolic acidosis in CKD	2 (6)	8 (25)	12 (38)	9 (28)	1 (3)
I have educational tools and resources available to help my patients understand:					
their CKD diagnosis	3 (9)	15 (47)	7 (22)	6 (19)	1 (3)
the potential medication-related risks associated with CKD	2 (6)	13 (41)	10 (31)	6 (19)	1 (3)
anemia of CKD^*^	2 (6)	6 (19)	13 (42)	8 (26)	2 (6)
hypertension in CKD	3 (9)	13 (41)	9 (28)	5 (16)	2 (6)
bone disorders in patients with CKD	2 (6)	6 (19)	14 (44)	8 (25)	2 (6)
hyperkalemia in CKD	3 (9)	6 (19)	13 (41)	8 (25)	2 (6)
metabolic acidosis in CKD	2 (6)	5 (16)	12 (38)	10 (31)	3 (9)

^*^n = 31;

^**^n = 30; Abbreviation: Chronic kidney disease (CKD)

### PCP-identified themes

The data were organized into major themes and subthemes by patient-, provider-, and systems-level barriers to CKD management, as well as potential facilitators of CKD management (Table 3).

**Table 3:** Primary care physicians’ perceived barriers and facilitators to CKD management.

**BARRIERS**	
**Major themes**	**Subthemes**
**Patient-level**	
Patients' limited understanding about CKD and its' implications	Patients with CKD are asymptomatic
	Patients often do not appreciate importance of CKD
	Patients' limited CKD knowledge and lack of symptoms contribute to decreased adherence with recommended treatment
Patients unable to afford recommended CKD care	Multiple medications, tests, and referrals for patients with CKD contribute to substantial health care costs
**Provider-level**	
PCPs' limited recognition or knowledge about CKD	PCPs may understand CKD less well than other areas of medicine
	CKD may not recognize CKD or add it to the problem list
PCPs' lack of awareness of CKD guidelines or useful algorithms for CKD care	Existing guidelines are not aggressively disseminated
	Existing guidelines are unclear
	Difficult to stay up-to-date with changing guidelines
CKD risk factors (blood pressure, diabetes, obesity) are difficult to manage	Blood pressure management is difficult
	Lack of patient access to self-monitoring tools (blood pressure monitoring kit)
	Conflicting treatment goals from different specialists
	Difficulty engaging patients in CKD self-management
PCPs' belief that they are unable to improve CKD	Belief that CKD is not reversible
**Systems-level**	
Limited visit time to care for complex patients	Healthcare system does not allow adequate time for management of complex patients
Poor reimbursement for delivering optimal CKD care	Limited reimbursement does not facilitate complex care
	More frequent visits may increase reimbursement but are unduly burdensome to patients
Lack of comprehensive clinical information systems (EMR)	EMRs lack sufficient flexibility for chronic disease management (e.g., patient registries)
Insufficient clinical support tools and resources to support patient-self-management	Inadequate patient educational material about CKD
	Lack of physician extenders, dietitians, educators, etc.
**FACILITATORS**	
**Major themes**	**Subthemes**
Decision support integrated into daily practice	Electronic care prompts
	Best practice / guideline support within laboratory reports
Automated eGFR reporting	Automated eGFR reporting to improve PCPs' CKD recognition
Team-based care	Better access to and utilization of dietitians, case managers, pharmacists, and health educators
Concise clear guidelines and CKD protocols	Useful guides to managing CKD as well as specific CKD complications (e.g., electrolyte abnormalities, metabolic acidosis)
Improved insurance coverage and reimbursement for CKD care activities	Improve compensation for CKD care
	Better align insurance coverage with clinical guidelines
Better CKD-related educational tools	Increase opportunities for PCP education in CKD
	Improve patient access to education and self-management resources
	Raise awareness of CKD within the general population

Abbreviations: Chronic kidney disease (CKD); primary care physician (PCP); electronic medical record (EMR), estimated glomerular filtration rate (eGFR)

### Patient-level barriers to CKD management

PCPs identified patient-level barriers to optimal management of CKD to be patients’ poor awareness and understanding of CKD, suboptimal adherence to treatment recommendations, and high burden of healthcare costs to patients. PCPs expressed that patients often do not recognize or understand the diagnosis of CKD, and are often surprised at the diagnosis given the frequent lack of attributable symptoms until late in the course of the disease:

*“They’re not expecting [it] and many of them don’t have relating symptoms*.*”*

PCPs felt patients’ lack of understanding of their CKD diagnosis and its implications on their health may adversely affect self-management of their CKD risks. A provider remarked,

*“Almost nobody is bothered by their chronic kidney disease until it’s way*, *way, way late stage, so they’re not particularly motivated [to manage their risk factors for CKD progression].”*

Physicians reported that since patients “*don’t feel”* their CKD, a discussion of CKD often leads immediately to patients’ fears of dialysis or transplantation, rather than a dialogue on chronic management. In this context, patient adherence to provider visits, diagnostic tests, medications, and lifestyle approaches to self-care does not meet PCP expectations. A PCP remarked:

*“You can order the tests*, *but …if they don’t understand why it’s important they’re not going to come in.”*

Out-of-pocket healthcare costs were also viewed as a barrier to patients’ adherence to provider recommendations:

*“To the compliance issue, if the patient is on multiple medications and seeing multiple specialists*, *so they have to balance cost. So a lot of time cost is an issue.”*

This concern was further reinforced in patients having to take time off from work to attend visits:

*“Somebody’s taken a day off of work to bring mom in who has otherwise no transport*, *so that person’s already out of work. Do you think they want to take another vacation day to come back in two weeks? No.”*

### Provider-level barriers to CKD management

PCPs identified numerous physician-level barriers in the management of CKD. They noted their lack of CKD knowledge, particularly regarding management of advanced CKD; poor awareness of CKD guidelines or difficulty implementing guidelines that are perceived to change frequently; and complexity of managing multiple comorbid illnesses with conflicting goals of care. Furthermore, they described a fatalistic belief that CKD is incurable and only likely to worsen, as well as difficulty engaging patients in modification of their CKD risk factors.

Some PCPs identified a lack of knowledge of CKD:

*“I feel like there's a lot of areas within medicine that I know a lot about…but renal…*.*It's not my super comfort zone”*

PCPs also described lack of recognition of CKD due to use of creatinine rather than estimated glomerular filtration rate (eGFR) or lack of documentation of CKD on the problem list as barrier to CKD management:

*“The lab did not calculate the GFR…I think that we probably missed a lot… [because] a creatinine 1.3…looks all right…*.*”*

*“If somebody's creatinine is 2.5 and you know its chronic kidney disease but that problem is not on the problem list, there's no flag…*.*that…triggers…the interaction….”*

Some PCPs had limited familiarity with CKD guidelines, while others perceived an absence of clear, easily applied algorithms for the management of CKD:

*“I know there’s like the National Kidney Foundation, but I feel like the ADA guidelines are much more useful… I mean I certainly don’t know them [CKD guidelines] very well and I can’t visualize an algorithm from them*.*”*

*“A good algorithm. I mean there are a lot of good algorithms for diabetes*; *there’s lots of good algorithms for hypertension. I haven’t seen a good algorithm for stage III kidney disease.”*

This perception of limited guidance from professional societies may be further exacerbated by recommendations that change over time with inadequate dissemination to practitioners:

*“And I think because a lot of those guidelines and rules change over time*, *there’s just a lot of confusion. So I think it is kind of this squishy black hole to a lot of primary care doctors as far as the nitty gritty details.”*

PCPs believed the management of patients with CKD was also complicated by the complexity of their medical issues and the difficulty in managing these comorbid illnesses such as hypertension, which sometimes is “*very hard to control*” later in kidney disease. Measuring blood pressure four times a year at an in-office visit was also noted as likely insufficient for best management. Managing comorbid illnesses of relevance to CKD progression was also perceived to be complicated by conflicting recommendations on goals of care based on other associated co-morbidities (e.g., blood pressure targets in patients with cardiovascular disease and CKD). A cardiologist, for instance, may be pleased when the blood pressure is lowered to 110/80, while the nephrologist is advocating for a higher target.

PCPs also described the struggles and frustrations they experience in engaging patients in self-management of risk factors for CKD progression. A provider stated:

*“What’s going to make them listen now if I’ve been doing that for the last 10 years before they got to the [CKD]? It’s a little bit frustrating*.*”*

Another provided commented:

*“It's a challenge to keep on top and keep your patients motivated to stay on the plan*.*”*

They also reflected that CKD is a difficult concept to explain and that during the visit they may place less emphasis on CKD compared to other conditions which may hamper patients’ recognition of the implications of CKD:

*“We're worrying about and thinking and monitoring their kidney disease but how much time are we really spending educating them and talking to them about it? Probably not that much. Which may then mean to them I guess I don't need to worry about it that much*.*”*

All of these provider-level barriers may be reinforced by a belief among some PCPs that CKD is incurable and unlikely to improve. A PCP commented:

*“If the blood pressure is high*, *I put them on blood pressure medicine, and I fixed it. If you have chronic kidney disease, you still have chronic kidney disease. You can’t fix it. All you can do is [ensure]… it doesn’t worsen. We’re not helping…it’s not very exciting.”*

When combined with PCPs’ perceived difficulty in engaging patients in their CKD care, PCPs may be further disincentivized to address the problem. Yet, some respondents also espoused a nuanced view of CKD management, suggesting that reframing expectations in CKD care may be required:

*“Maybe the excitement will come 25 years from now as far as this patient never getting to dialysis*. *It’s sort of a delayed gratification… This one might be just very, very delayed.”*

### Systems-level barriers to CKD management

In addition, PCPs believed that inefficiencies in the health care system hindered CKD management. These challenges included insufficient time for managing a complicated disease process such as CKD in patients who often have multiple medical problems, as well as limited reimbursement, rigid electronic medical record (EMR) templates, and inadequate clinical support and resources. PCPs focused heavily on time constraints within a healthcare delivery system that is not optimized for the management of patients with multi-morbidity, nor the management of a multi-faceted disease process such as CKD. As care of the CKD patient necessitates patient education and management of hypertension, diabetes, anemia, and other comorbid illnesses or acute concerns, there is often insufficient time in a clinic visit to address all relevant medical and behavioral topics. Thus, it is easy to deprioritize CKD or defer management until another visit:

*“I think during the 15 or 20 minutes you have with the patient appointment*, *your agenda’s long. You need to deal with their blood pressure and their diabetes and they may come in because their back’s hurting or something else.”*

*“The patient is not symptomatic*, *the patient is not going to complain about it [CKD], so you worry about the sore throat for the day or the cholesterol and so on.”*

PCPs identified insufficient reimbursement as a barrier to providing better care. While they noted increased reimbursement for more frequent visits, a lack of appointment availability was identified as a limiting factor. In addition, PCPs indicated that not all laboratories automatically report eGFR to facilitate recognition of CKD; EMR systems were not optimized for chronic disease management; adequate educational resources were not available (e.g., patient educational material for non-English speaking patients), and many practices lacked physician extenders to facilitate longitudinal care and patient education.

### Facilitators of CKD management

Respondents also proposed numerous facilitators to address these barriers to CKD management. PCPs desired decision support (e.g., electronic prompts with EMR or laboratory-based), automatic eGFR reporting by all laboratories, more robust team-based care, concise and clear CKD guidelines, increased compensation and better insurance coverage for CKD care activities, and better CKD-related educational tools to facilitate CKD management. PCPs felt it would be helpful to have prompts integrated in their daily practice to remind them of CKD related care activities. For PCPs who had decision support within their EMR or through written guidance that accompanied laboratory reports, they found it to be extremely helpful in facilitating CKD care in accordance with guidelines. A PCP described,

*“Our EMR has lots of pop-ups that tells exactly what to order and that’s helpful*.*”*

PCPs also felt that automated eGFR reporting enhanced their recognition of CKD, and felt it important that all laboratories universally report eGFR when a creatinine is ordered.

PCPs desired greater use of robust multi-disciplinary care teams, such as dietitians, case managers, pharmacists, and health educators to facilitate patient education and self-management of risk factors for CKD progression. Those PCPs who had access to these resources found them extremely helpful:

*“It does help as we shift more towards population management and value over volume–medical home as a model becomes key in chronic disease management*.*”*

Respondents believed ready access to clear and concise CKD guidelines was needed, including for more challenging nephrology topics such as electrolytes and metabolic acidosis:

*“If it were a really practical guide–which I have never seen personally–to managing those electrolyte complications, I think that would be incredibly helpful*.*”*

In addition, improved reimbursement for CKD care activities and alignment of insurance coverage with those clinical guidelines to remove potential financial barriers for patients was deemed important for effective CKD care delivery:

*“I think if we were compensated better for the management of these chronic complex problems we could actually spend more time with the patient and it wouldn't be as much of a challenge*…*”*

*“I find lately*, *and probably the last 10–15 years, insurance guidelines actually trump the clinical guidelines. And it’s unfortunate, but you cannot fight them, and you cannot explain to the patients–this is what the nephrologist’s association says, but this is what your insurance says. Where do you go from here?”*

PCPs also desired additional educational resources to enhance their understanding of CKD management (e.g., web-based tools and/or CME activities) as well as greater access and use of effective education tools to facilitate patient CKD education. Ultimately, raising awareness of CKD within the general population was believed to be critical. “*More media coverage*” and “*even public service announcements*” might increase patient engagement in their CKD management, and this might be further facilitated by dedicating visits specifically to CKD discussion.

In comparing the PCPs responses on the questionnaire with the themes identified in the focus group discussions, the findings were similar. In both questionnaire and the focus groups, physicians report lack of awareness and familiarity with CKD guidelines, limited comfort with managing CKD (particularly CKD related complications), and lack of available resources to facilitate optimal management of CKD or to improve patients’ self-management of their risk factors for CKD progression. There were no obvious contradictions between the questionnaire and focus group findings; however, the focus group provided more comprehensive understanding of the challenges providers were facing. For example, while the majority of providers reported feeling comfortable with the diagnosis of CKD (94%) on the questionnaire, in the focus groups, PCPs reported challenges with follow-up steps required to deliver optimal care such as quantifying the severity of CKD or documenting it on the problem list so it can be addressed routinely during subsequent follow-up visits. A provider commented:

*“I know what the diagnosis is*. *The challenge is what stage is this or documenting the diagnosis.”*

## Discussion

In this study, PCPs identified numerous challenges to providing optimal CKD care, including patient- (e.g. patients’ limited understanding of CKD and inadequate adherence to recommended treatment), physician- (e.g., PCPs’ limited familiarity with CKD guidelines, difficulty with managing risk factors for CKD progression, and belief that they are unable to improve CKD), and systems-level (e.g., limited visit time to care for complex patients, lack of comprehensive clinical information systems to track and plan care; and insufficient resources to support patient self-management of their CKD risks) barriers. They also identified facilitators that may provide solutions to some of these problems (e.g., concise clear guidelines and protocols, decision support integrated into the EMR, and team-based care).

The findings from this study are consistent with other studies that have used various methods (e.g., questionnaires, medical record review, interviews or focus groups) to characterize PCPs practice patterns and identify barriers to the management of CKD by PCPs.[9, 11, 13, 16–23] However, there are few studies that explore in-depth the reasons for ineffective CKD care by US PCPs, whose experience in delivering CKD care may differ from other countries with universal health care coverage.[9, 13, 16] This study builds on the existing evidence to provide detailed views of the challenges US PCPs’ experience in caring for patients with CKD. The findings from this study provide valuable insights that can be used to improve delivery of CKD care in primary care settings in the US, and that may also be generalizable to PCPs practicing in other countries or caring for other conditions.

Patient-level barriers identified by the PCPs in our study included limited awareness and understanding of CKD, inadequate adherence to treatment recommendations, and healthcare costs. These findings are consistent with prior studies describing patients’ poor understanding of CKD and its health implications.[24–28] A central tenet of contemporary medicine is that informed patients have better health outcomes.[29] In CKD, however, studies have demonstrated that knowledge of CKD among patients is quite low relative to other common chronic diseases such as diabetes or hypertension.[24–28] CKD is conceptually difficult for patients to understand, and effective management often requires significant financial cost and substantial changes to diet and lifestyle to mitigate risks for CKD progression.[30, 31] PCPs can play a key role in improving patients’ CKD awareness and self-management behaviors. Although the majority of PCPs in our study reported feeling comfortable educating patients about CKD, on further questioning they expressed challenges in improving patients’ understanding of their CKD diagnosis and engaging them in risk factor modification. These findings emphasize the need for greater dissemination of tools and resources to facilitate patient education about CKD and to enhance patients’ understanding of CKD risks.[32] Linking CKD education to other co-existent medical conditions, such as diabetes and hypertension, may enhance patients’ CKD awareness and also facilitate increased delivery of CKD education during time constrained visits.[27] Greater access to effective self-management support within primary care (e.g., case management, community health workers services, referral to evidence-based self-management education programs, caregiver education and support) should also be an integral component of strategies seeking to improve patients’ CKD awareness, treatment adherence, and achievement of CKD care goals.[33–35] While such interventions have proved successful in other diseases, a recent randomized trial of patient navigators and enhanced personal health records in CKD failed to demonstrate a meaningful impact on multiple CKD outcomes.[36] The study, however, may have been underpowered and high risk patients were not specifically targeted. Additional work in this area is needed.

The PCPs in our study also identified several of their own challenges in caring for patients with CKD, including limited CKD knowledge, less familiarity with CKD guidelines, and difficulty in managing the complexity of multiple comorbid illnesses. A belief that CKD is incurable may also reinforce these barriers. Nephrology is frequently cited as a difficult topic area by trainees pursuing subspecialty training in disciplines other than Nephrology.[37] Deficiencies in CKD knowledge relative to other topics such as diabetes mellitus have been identified in Internal Medicine residents in-training.[38, 39] In addition, among 651 surveyed residents, 28% were unaware of the existence of CKD guidelines at a time when the Kidney Disease Outcomes Quality Initiative (K/DOQI) guidelines had been published for 5 years.[40] Since then, the K/DOQI guidelines have largely been supplanted by multiple, lengthy consensus documents from the Kidney Disease Improving Global Outcomes (KDIGO) consortium. KDIGO guidelines are targeted to nephrologists, and not surprisingly, robust implementation by PCPs has been limited in both the United States and elsewhere.[19, 41, 42] This is consistent with findings from our study, where 45% of PCPs reported not following CKD guidelines. Furthermore, most of the PCPs in our study reported feeling comfortable managing their patients with CKD, but less comfortable managing CKD-associated complications (e.g., metabolic acidosis, bone disorders, or anemia). This suggests PCPs may be comfortable with the premise of managing patients with CKD but are less well-prepared to actually implement guideline specific therapies.

Clear and concise CKD guidelines are needed to facilitate greater implementation by PCPs. To help address CKD knowledge gaps and to increase recognition and utilization of CKD guidelines, both the National Kidney Foundation (NKF) and the American College of Physicians have published CKD algorithms targeted to PCPs.[43, 44] In addition, NKF has launched CKD*inform*, a collection of evidence-based resources and protocols, to educate PCPs on best practice in early-stage CKD management.[45] Greater dissemination and uptake of these resources within primary care and enhanced early education of primary care trainees in the key aspects of CKD management may improve PCPs provision of guideline-concordant CKD care.[9] In addition, since PCPs often care for patients with multiple medication conditions, these strategies should aim to reconcile any discordant treatment goals when managing CKD in the context of multi-morbidity. Opposing treatment goals could increase the risk of death, hospitalization, and emergency department visits in this population.[46]

Furthermore, systems-level barriers to PCPs providing CKD care included insufficient time for managing complicated disease processes, limited reimbursement, rigid EMR templates, and inadequate clinical and educational resources. Computerized decision support systems have been increasingly implemented within electronic health records to facilitate delivery of guideline concordant care, and may be particularly useful among PCPs who are often charged with managing patients’ multiple and/or complex medical conditions during brief clinical encounters. [47] The literature, however, demonstrates mixed results to date on the impact of EMR and clinical support tools in CKD management. The introduction, for example, of automated eGFR reporting has been associated with increased identification of CKD, but only marginal improvements in care as measured by percentage of late referrals to Nephrology or prescription of renin angiotensin aldosterone system antagonists.[48–50] Similarly, clinical decision support systems have resulted in only small improvements in physician adherence to CKD guidelines.[51–54] Although desired by most PCPs, additional work is needed to understand how best to implement electronic support tools to meaningfully impact patient care and outcomes.

In addition to clear and concise guidelines and decision support, PCPs desired more robust implementation of patient-centered medical homes, and/or greater ease in accessing multi-disciplinary care teams that include dietitians and pharmacists. Currently, PCPs report a lack of resources and infrastructure to support effective CKD care. Value-based care models such as accountable care organizations (ACOs) and Patient-Centered Medical Homes have the potential to transform care delivery and provide PCPs with additional resources to provide optimal CKD care, particularly for patients with multiple comorbidities. Given the complexity of CKD, increased partnerships between PCPs and other providers may also facilitate more efficient and effective CKD care delivery. To this end, a recent randomized trial demonstrated small improvements in medication management in patients with CKD when community pharmacists received focused training in CKD.[55] Broad implementation of such programs that facilitate delivery of optimal CKD care, however, remains challenging in light of time and financial pressures. Results from technology-based intervention trials such as the Kidney Awareness Registry and Education (KARE) study, a study evaluating the effectiveness of an electronic CKD registry and self-management support program that consists of automated telephone modules and health coaching, may ultimately inform potential solutions for the primary care setting.[56] As such, interventions that combine patient education and self-management support with resources that facilitate provision of optimal CKD management within primary care will be critical to addressing the multiple barriers voiced by PCPs. Ultimately, efforts to raise awareness of CKD in the social conscious–as has been achieved with cardiovascular disease and breast cancer, for example–may help physicians and patients find shared goals. Given the challenges, it would be reasonable for PCPs to steer resources toward those patients at highest risk for CKD progression or related events such as cardiovascular disease.[57–61] Population risk stratification by eGFR and albuminuria are the best clinically available metrics, although utilization remains low.[41, 62]

The limitations of this study deserve mention. The sample size is small and the perspectives of the physicians in this study may not represent all of the practice challenges across the diversity of primary care providers (including nurse practitioners and physician assistants) or practice settings. However, our study included participants representing diverse sociodemographic and practice characteristics. Second, our study targeted primary care physicians. Focus groups with patients may also provide additional insights regarding barriers to caring for patients with CKD in primary care settings.

In conclusion, PCPs identified multiple modifiable patient-, provider-, and systems-level barriers to the optimal care of patients with CKD. These physicians also identified potential facilitators to providing better CKD care in the primary care setting. To improve the care and clinical outcomes of patients with CKD and achieve Healthy People 2020 CKD objectives, efforts are needed to improve dissemination of tools and resources that can be implemented in primary care to facilitate improved patient awareness of CKD.[63] Improving patients’ access to self-management resources to further help mitigate their CKD risks is also needed. Additionally, interventions and health care delivery innovations that enhance PCPs’ CKD knowledge, facilitate PCPs’ provision of guideline concordant care, and/or integrate multidisciplinary teams may improve PCPs’ capacity to effectively care for patients with CKD.

## Supporting information

S1 Appendix PCP focus group survey. (PDF)
