# Supplementary material for: Primary care physicians’ perceptions of barriers and facilitators to management of chronic kidney disease: A mixed methods study
Source: PLoS One. 2019 Aug 22;14(8):e0221325. doi: 10.1371/journal.pone.0221325 (PMC6705804; doi:10.1371/journal.pone.0221325)
Supplement: S1 Appendix — (PDF) [file pone.0221325.s001.pdf]

Provider Survey

SID_________

This document consists of two sections: 1) demographic survey and 2) CKD practice survey. We appreciate your taking the time to complete both sections.

Section 1: Demographic Survey

1- What is your age? _____

2- What is your gender?
____ Male
____ Female

3- Are you Hispanic or Latino?
____ Yes
____ No

4- What is your race? (please check one)
____ American Indian or Alaskan Native
____ Asian
____ Black or African American
____ Native Hawaiian or Other Pacific Islander
____ White
____ Multi-racial
____ Other, please specify__________________

5- What is your medical specialty? (please check one)
____ Internal medicine
____ Family practice
____ General practice
____ Med/Peds
____ Other, please specify______________________

6- What best describes your training? (please check one)
____ MD
____ DO
____ Nurse Practitioner (NP)
____ Physician Assistant (PA)
____ Other, please specify_________________

7- Which of the following best describes your practice setting? (please check one)
____ Solo private practice
____ Single specialty group private practice
____ Multispecialty group practice
____ Health maintenance or staff model
____ University hospital or medical school
____ Community, teaching hospital
____ Community, non-teaching hospital
____ Government health care facility

____ Other, please specify_________________

8- What percent of your time is currently spent performing each of the following duties?

a. Clinical………………. _______%
b. Research…………… _______%
c. Administrative……… _______%
100%

9- Including you, how many providers are in your main practice? (please check one)

____ 1
____ 2 – 5
____ 6 – 10
____ 11 – 50
____ More than 50

10- What is the zip code of your primary practice location? ___ ___ ___ ___ ___

11- How many patients do you see per week? (please check one)

____ 50 or less
____ 51 – 100
____ 101 – 150
____ 151 – 200
____ More than 200

12- How many CKD patients do you see per week? (please check one)

____ 10 or less
____ 11-20
____ 21-30
____ 31-40
____ More than 40

13- How many years have you been in practice? (please check one)

____ 0 – 5
____ 6 -10
____ 11- 15
____ More than 15 years

14- Do you use electronic health records (EHR)? (please check one)

____ Yes, all EHR
____ Yes, part EHR and part paper
____ No

15- Do you follow any chronic kidney disease guidelines? (please check one)

____ Yes
____ No

If yes, which guidelines: ………………………..

16- What proportion of your patients are covered by the following types of insurance ? (please check one)

____ Medicare

____ Medicaid

____ Private

____ Uninsured or self-pay

____ Other, please specify__________________

Section 2: CKD Practice Survey

Instructions: Please read each statement. Check the box in the column that describes your level of agreement with the statement.

Statements	Strongly Agree	Agree	Neutral	Disagree	Strongly Disagree
**I feel comfortable:**					
Making the diagnosis of chronic kidney disease (CKD) in my patients					
Educating my patients about CKD					
Managing my patients with CKD					
Managing medication dosing in my patients with CKD					
Avoiding nephrotoxic medications (e.g., NSAIDs) in my patients with CKD					
Managing hypertension in my patients with CKD					
Managing anemia of CKD in my patients					
Managing bone disorders of CKD in my patients					
Managing electrolyte disorders (e.g., hyperkalemia) in my patients with CKD					
Managing metabolic acidosis in my patients with CKD					
**I have available tools (e.g., electronic medical record alerts; checklists; or printed, web-based, or smartphone-based resources) which help me to:**					
Diagnose CKD					
Manage CKD					
Manage medication dosing					
Avoid prescribing nephrotoxic medications					
Manage hypertension in my patients with CKD					

Manage anemia of CKD					
Manage bone disorders of CKD					
Manage hyperkalemia in CKD					
Manage metabolic acidosis in CKD					
**I have educational tools and resources (e.g., printed and web-based materials/programs, classes, or health educators) available to help my patients understand:**					
Their CKD diagnosis					
The potential medication-related risks associated with CKD (e.g., nephrotoxins, medication dosing)					
Anemia of CKD					
Hypertension in CKD					
Bone disorders in CKD patients					
Hyperkalemia in CKD					
Metabolic acidosis in CKD					
